# What women want to hear: the helpful and unhelpful comments reported by women struggling with infertility amidst the COVID-19 pandemic

**DOI:** 10.1371/journal.pone.0318921

**Published:** 2025-02-25

**Authors:** Ashley A. Balsom, Loveness Dube, Jennifer L. Gordon

**Affiliations:** 1 Department of Psychology, University of Regina, Regina, Saskatchewan, Canada; 2 Department of Psychology, Memorial University of Newfoundland and Labrador, St. John’s, Newfoundland and Labrador, Canada; The University of Sydney, AUSTRALIA

## Abstract

**Introduction:**

Many women struggling with infertility report that they frequently experience unhelpful social interactions with well-meaning loved ones and healthcare providers, contributing to a reluctance to confide in others about their infertility and emotional distress. However, it remains unclear what interaction content women experience as ‘helpful’ versus ‘unhelpful,’ making it difficult to provide concrete recommendations to the public about how best to support individuals struggling with infertility.

**Methodology:**

Eighty women from Canada and the United States (ages 20–45 years) whose fertility treatments had been cancelled due to the COVID-19 pandemic were recruited via social media to complete an online survey, which included two open-ended questions about the most helpful and unhelpful social interactions they had had about their infertility. Two independent researchers conducted content analysis to identify categories of helpful and unhelpful social interactions.

**Results:**

The following six categories were identified by women as helpful: 1) Listening, 2) Fostering hope, 3) Talking to individuals with lived experience, 4) Distraction, 5) Validating emotions, and 6) Tangible support. Responses about unsupportive interactions fell into four categories: 1) Toxic positivity, 2) Advice-giving, 3) Invalidation, and 4) Intruding. Sample quotes from each category are provided.

**Conclusion:**

These findings provide valuable insights that can be used to develop future educational materials for the general public on how to interact with individuals experiencing infertility.

## Introduction

Approximately one in six couples of reproductive age faces infertility, traditionally defined as the inability to achieve conception after 12 or more months of unprotected intercourse or an impairment in a person’s reproductive capacity individually or with their partner [1]. Despite the issue being equally likely to originate from either partner, women^1^ often bear the responsibility of seeking information and undergoing fertility testing and treatment [2] and are disproportionately burdened with an array of psychological difficulties [3–5]. Half of women with infertility endorse it as the most upsetting experience of their lives [6].

Those grappling with infertility seek emotional support from various sources, including parents, partners, friends and anonymous peers through online platforms [7–9]. When the support received is perceived as comforting and helpful, it has been found to benefit the mental health of those impacted by infertility [10–13], with individuals reporting more social capital exhibiting better emotional adjustment to infertility [14–16]. Supportive social interactions in the context of infertility have even been found to lower levels of the stress hormone cortisol [17].

However, women with infertility report varied experiences with social support from loved ones [7]: while some report being met with understanding and empathy, a significant lack of understanding and sensitivity from loved ones is also commonly reported, resulting in social support that is experienced as more harmful than helpful [7,18]. One study found that individuals struggling with infertility often face unhelpful and hurtful comments from others due to a lack of understanding and experience [19]. Research has associated unsupportive social interactions with poor psychological outcomes [3–5]. Unhelpful comments from loves ones can also perpetuate infertility stigma and exacerbate infertility-related stress experienced by individuals. These comments can further exacerbate the emotional distress experienced by individuals dealing with infertility. Receiving unhelpful comments from others may also lead individuals to retreat from their social networks, in turn contributing to a lack of understanding from others and further hampering others’ ability to provide effective emotional support [20]. Additional research regarding the specific comments that are experienced as upsetting rather than comforting in this context may be helpful in developing educational resources focused on how the loved ones of those struggling with infertility can provide more effective support.

The emotional toll of infertility was considerably amplified by the onset of the COVID-19 pandemic, marked by the temporary suspension of all in-person fertility treatments. This precautionary measure, recommended by the American Society of Reproductive Medicine and the Canadian and Andrology Society on March 17^th^, 2020 [21,22], not only led to the delay of new treatment cycles but also the abandonment of ongoing treatment cycles. Although the complete suspension of in-person treatments lasted just over a month, the resumption of services was gradual, resulting in prolonged delays for treatment-seeking patients. A survey conducted by our team revealed a considerable negative impact on the mental health of those impacted by the suspension [23], and subsequent research has further affirmed these findings [24–27].

Our previous study on the psychological repercussions of fertility treatment suspensions identified a positive association between perceived social support and mental health amidst treatment suspensions [23]. However, that report did not examine the types of comments received by loved ones related to treatment suspensions, though this information was collected in that survey. Specifically, participants were asked to report the helpful comments they had received from loved ones amidst COVID-related treatment suspensions, as well as those experienced as unhelpful. The purpose of the current report is to explore relationships among comments received by loved ones amidst COVID-related treatment suspensions perceived as helpful or unhelpful. Understanding the frequency of endorsing specific categories of support, could help us understand not only the types of support commonly offered but also the patterns of social interaction that participants perceived as helpful versus unhelpful. By examining frequency, we aim to identify prevalent communication approaches that may influence wellbeing, as well as to highlight which types of comments are most frequently associated with positive or negative experiences. This information can inform support strategies and implementation science strategies by emphasizing types of comments that may be more universally helpful and those that could unintentionally contribute to distress.

## Methods

As part of our larger study examining coping strategies and the perceived impact of infertility treatment suspension on psychological well-being, we recruited women from across Canada and the United States to participate in the online study via advertising on Facebook between May 16th and June 13th, 2020, as previously described [23]. To qualify, women had to live in Canada or the United States and report having had their fertility treatments suspended due to the COVID-19 pandemic and that their treatments had not yet resumed. The study ad instructed prospective participants to message the research team if they were interested. A member of the team then verified their eligibility to participate. If deemed eligible, they were provided with a link and password to access an online survey using the survey software Qualtrics. Participants provided informed written consent by clicking on “agree to participate” at the bottom of the consent form displayed at the start of the online survey. This consent form outlined the study’s purpose, procedures, potential risks and benefits, confidentiality, and the voluntary nature of participation, ensuring that participants were fully informed before agreeing to take part in the study. Individuals were compensated with a $15.00 Amazon e-gift card for their participation. The study was approved by the University of Regina Research Ethics Board (REB #2020-061).

A purposive sampling method was used to reach a large and diverse sample of individuals whose fertility treatments had been cancelled in either Canada or the United States. This methodology was decided upon due to variability in treatment delays across states/provinces and individual clinics, as well as variability in wait times experienced by individual patients, depending on the stage their treatment stage when the pandemic hit.

After completing quantitative surveys, results of which have been previously published [23], participants were asked two open-ended questions: 1) “What were some of the most helpful things people said to you to help you cope with your fertility struggles?” and 2) “What were some of the most unhelpful things people said to you to help you cope with your fertility struggles?”. Data from the open-ended questions was exported into Excel for data extraction. Content analysis was conducted independently by the first and second authors, and the third author helped to resolve disagreements.

We used an inductive manifest content analysis to systematically analyze participants’ responses. This approach focuses on identifying and quantifying explicit, surface-level content, allowing categories to emerge directly from the data without preconceived categories. This inductive process aligns with our aim of capturing participants’ actual words and perspectives, making it a suitable method for analyzing open-ended survey responses where exploratory insights are sought [28].

To ensure rigor, the first and second authors independently coded the responses of 20 participants. This initial coding phase allowed each coder to identify preliminary codes based on manifest content (i.e., content directly expressed by participants). After coding this subset, both authors met to discuss and refine these initial codes, resolving any discrepancies and enhancing consistency and reliability in their coding approach. This collaborative process led to a finalized coding scheme, which was subsequently applied to the remaining data. The coders were a post-doctoral fellow and doctoral student in Clinical Psychology in the Reproductive Mental Health Research Unit with a research specialization in the area of infertility. The interrater reliability of the coding was 93% for the helpful comments and 95% for the unhelpful comments, resulting in an overall interrater reliability of 94%.

An inductive coding technique was utilized to identify categories for the textual data.

Manifest content analysis is effective in providing a structured yet flexible approach to understanding explicitly content in participants’ responses, thus aligning with our research objectives. Content analysis is also widely regarded as appropriate for analyzing open-ended survey data [29–31], allowing us to systematically quantify content without inferring underlying or latent meanings [32]. We decided to include coding frequency to provide additional insights into the extend of endorsement of each category. Reporting frequency helping to illustrate topics and perspectives that were more commonly expressed by participants, offering a clearer sense of shared experiences or widely endorsed sentiments.

## Results

### Participants

Though 92 participants completed the quantitative portion of the survey, only 80 participants responded to one or both of the open-ended questions on helpful and unhelpful interactions. Of these 80 participants, six answered only one of the open-ended questions; the answer that they did provide was included in the coding. Among participants included in the qualitative analysis, ages ranged from 20 to 45 years, with a mean of *M(SD)* =  34.2 (4.8). Time spent trying to conceive ranged from 5 to 180 months, with a mean of *M(SD)* =  37.6 (33.7). In terms of the types of treatments that were canceled, 70% had had an IVF cycle cancelled, and 30% were in the midst of IUI. Seventy-four percent of participants were White, 3% Black, 8% Latina, 13% Asian, and 2% other. Most women were experiencing primary infertility, with 40% of participants having one biological child. Ninety-five percent of participants were married or in a common-law relationship. Fifty-three percent of participants were from Canada, and 47% were from the United States. In terms of the highest level of education achieved, 10% had a high school diploma, 18% had completed some university, 44% held a bachelor’s degree, 21% held a master’s degree, and 8% held a doctorate. Concerning income, 60% of the sample made $90 000 a year or greater.

### Identified categories

#### Helpful comments.

Reported helpful interactions fell into six broad categories (Table 1). The category of “Listening” represents having a loved one simply listen to the woman’s frustration and pain, without interruption or offering advice. Participants endorsed that this was helpful in coping with the cancellation of treatments resulting from the COVID-19 pandemic. As one participant reported: “The most helpful thing is to just listen. I can’t even decide where I stand now that treatments are in limbo, so just let me cry and complain.” Most comments under the category of “Fostering hope” related specifically to the hope that the pandemic would be over soon and that treatments would resume soon enough. Some focused on a specific aspect of treatment: “It will happen in time, that technology is advancing and there will be a way to conceive once the pandemic is over” or referred to their spiritual beliefs “It’s going to happen when the time is right. Put it in God’s hands”. The third category related to a shared understanding with other women in a similar situation. Participants noted that they found it helpful to talk with someone who had been through fertility treatments as well (e.g., “shared their struggles,” “talked with friends who did gestational surrogacy,” “[shared] that they are going through the same thing”). The fourth category related to encouraging distraction for women whose fertility treatments had been cancelled. This category included things such as focusing on other aspects of their life such as their own health: “this is an opportunity to improve my health more before IVF again” and self-care “focus/spoil yourself”. The fifth category related to receiving validating and empathic statements about the pain they were experiencing. Specifically, women reported that they found it helpful when their experience was acknowledged and validated, with responses such as “it’s understandable to feel the way you do” and “it’s okay to feel this way.” Finally, women also appreciated that their loved ones offered tangible support, such as in the form of financial help for fertility treatments.

**Table 1:** Categories and sample quotes of helpful and unhelpful interactions.

Categories	Endorsement	Sample Quotes
Helpful Comments		
Listening	35%	“Just let me cry and complain”
		“That they’re here for me if I want to talk”
Fostering hope	35%	“This isn’t going to last forever”
		“You will enjoy life again, even if it’s without kids. Life can be full and fulfilling without kids.”
		“This is just a temporary situation and the treatments would be able to be started again in the not too distant future.”
Sharing lived experience	13%	“That they are going through the same thing.”
		“What was helpful was connecting with other people in the same boat. I fully believe that distancing & lockdown efforts are important. But I also find it difficult to deal with yet more delays to my fertility process.”
Encourage distraction	8%	“Give me idea of what to do to keep me busy, try to keep my mind off of it”
		“He … helped me see that we could try to enjoy the forced ‘break’ from fertility treatments to focus on us as a couple”
Validation	6%	“It’s a rough situation and that pregnancy is one of those things that it doesn’t matter how hard you try. Hard work does not equal success. And that it’s not my fault and that I’m doing my best.”
		“That my feelings are valid. It’s ok to be sad/disappointed/angry about the situation.”
Offer of tangible support	3%	“My parents offered financial help for a surrogate if this next round doesn’t work. It takes some of the pressure off”
		“How can I help?”
**Unhelpful Interactions**		
Toxic Positivity	41%	“don’t worry! you’re still so young! A year wait wouldn’t really be so bad, you can enjoy other things!”
		“Everything happens for a reason. Maybe you’ll get lucky and have a quarantine baby! It’ll all be ok in the end.”
Giving advice	28%	“You are trying too hard. Just relax and it will happen”
		“I heard that if you try (insert something random) that will help and well you could always adopt”
Invalidation	24%	“Stop complaining…people have it worse”
		“You had one kid, and you have step kids, isn’t that enough?”
Intruding	7%	“Constant questions about everything or being over invasive with questions and wanting too much detail”
		“Coworkers asking when I’m going to have a baby”

Apart from the above-identified categories, two comments reported by participants did not fall into these identified categories, including “it’s not a good time to have a baby anyway,” and “you are stronger than you think.” Eight participants also reported that there was nothing that people can say that could have helped them or that would have been comforting in those moments.

Of the helpful comments, ten were explicitly linked to the pandemic or treatment interruptions, such as reassurances that “COVID can’t last long. It’ll be over before you know it,” “My husband keeps saying that it isn’t permanent. Eventually, we will be able to pursue IVF. The pandemic won’t last forever”. The remaining 67 helpful comments were more broadly about infertility, without specific reference to the pandemic.

#### Unhelpful comments.

Unhelpful comments were categorized into four categories (Table 1) based on responses from 77 participants. The category “Toxic Positivity”, reflected loved ones’ tendency to insist that the woman with infertility maintain a positive attitude and avoid all negative emotions. Statements such as “Just stay positive”, and “Look on the bright side” are prime examples of ‘toxic positivity’. This also includes saying things such as “it will happen”, “everything happens for a reason,” or “it will all work out.” One participant described this type of unhelpful comment as “naïve confidence that it will work out,” which appears to be an adequate summary of the difficulty women experience with toxic positivity. Some statements falling under this category had religious undertones: “That it is all in God’s hands and that I should trust his timing. (I believe in science and am not religious.)”. This underscores the importance of tailoring expressions of hope to the individual’s unique perspective and preferences. The second most commonly endorsed category related to receiving unsolicited advice on how to conceive without medical assistance or become a parent through alternative means, for example: “You can always adopt,” “just relax and it will happen,” and “drink some alcohol and go have fun…that’s how I got pregnant”.

The third category referred to remarks minimizing or discounting the validity of the woman’s pain. This included comments such as “you won’t be the first or last person not to have a child,” “stop complaining, people have it worse,” and “having a baby is horrible … be thankful you can’t”. Finally, some women did not appreciate when others would pry for details about their struggles to conceive. These women described statements such as “kept asking if I had heard anything yet,” “asking if I was pregnant,” and “after miscarriage – will you try again?” Advice-giving and making invalidating statements are frequently devoid of evidence. Advice-giving statements, such as “just relax, and you’ll get pregnant”, “why don’t you just adopt”, and “have you tried acupuncture?” lack an understanding of the extensive research and efforts individuals have invested in their fertility journey.

Only three unhelpful comments were specifically related to the cancellation of treatments due to COVID-19, including “Your body probably needed a break anyway”, “Everyone is going through the same thing”, and “You don’t want to be pregnant during COVID anyways”. All other unhelpful comments were more generally about the experience of infertility unrelated to the pandemic. There was one comment that did not fit within the identified unhelpful categories “That they don’t really know what to say because they can’t help me. Often not saying much at all makes it seem so much more real which sucks.”

## Discussion

Women with infertility commonly recount negative social interactions with loved ones, acquaintances, and health professionals alike [5,33,34]. This underscores the pressing need for widespread education on infertility and effective ways to support individuals navigating this challenging journey. The findings from our current study contribute to fulfilling this need by clearly identifying the types of comments that women facing infertility find either helpful or unhelpful, particularly amid the profound stress of fertility treatment suspensions during the COVID-19 pandemic. However, our results also highlight the potential pitfalls of unintentionally making unhelpful remarks, even with the best intentions. Notably, many quotes illustrating unhelpful comments appeared to be well-intentioned. In the ensuing discussion, we delve deeper into the identified categories, offering concrete recommendations and emphasizing crucial distinctions between seemingly similar yet distinctly helpful and unhelpful approaches to providing social support. The purpose of the current report is to enhance understanding in infertility-specific social interactions by exploring relationships among comments received by loved ones amidst COVID-related treatment suspensions perceived as helpful and unhelpful.

Despite the pressure loved ones may feel to ‘say just the right thing’ to someone struggling with infertility, our findings underscore that the most appreciated form of support is often simply being listened to without interruption. This aligns with research on other chronic health conditions, such as HIV and chronic pain, where having a support person respond with empathic listening has consistently shown associations with better mental health outcomes [35,36]. This may help relieve the pressure on loved ones who are unsure what to say. These results suggest that loved ones can extend a straightforward “I’m here to listen anytime you want to talk.” When the person is ready to share, using active listening skills (e.g., turning off distractions, providing undivided attention, asking non-intrusive follow-up questions) can ensure the person feels truly heard. This approach reduces the burden on loved ones who may struggle to find the right words and allows the individual with infertility to have agency on when they may be ready to receive this support. Extending an offer to listen anytime and allowing the person with infertility to approach when they’re ready may also help guard against “intrusion” – one of the identified unhelpful interactions. The demonstration of active listening skills and empathy by loved ones has been demonstrated relatively consistently throughout the literature to effectively promote mental wellness in various populations, such as individuals with dementia, those coping with cancer, and in the context of marital relationships [37–40]. Research furthermore suggests that the benefits of active listening may be particularly evident in the context of highly stressful life events [41–44], consistent with its benefits in response to fertility treatment suspensions.

Similarly, being offered reasons to be hopeful emerged as a highly endorsed supportive interaction. However, caution is advised in using this strategy due to its similarity with a highly endorsed unhelpful strategy–‘toxic positivity’. This harmful approach involves outright rejection of all negative emotions – disappointment, frustration, grief – no matter how difficult the circumstances [45]. This can be dismissive of genuine pain experienced by the individual. When offering reasons to be hopeful, it is essential to create space for the person to experience and express their ‘negative’ emotions without judgment. Something like, “It’s completely understandable that you’re frustrated and disappointed right now. I know it’s hard, but there will be an end to all this, and you will be back in treatment one day.” One would want to avoid giving the impression that what is really intended is “stop your complaining already and just wait for this to be over.” Balancing the provision of reasons to be hopeful with active listening and validating statements can help avoid conveying an unintended message of impatience or dismissal.

Furthermore, it’s crucial to be attentive to the person’s response when offering reasons for hope and be mindful of potential signs that this may not be what they need at that moment. One important consideration is mentioning spirituality. While some women may find it helpful to be offered hope with their spiritual connections, such as “look to God,” this may be unhelpful to those who are not religious, and they may find this statement invalidating. It seems reasonable to consider the relationship the person has with spirituality before making this type of statement.

Women with infertility also reported finding it helpful when their support systems encouraged them to focus on aspects of life unrelated to their fertility. Indeed, engaging in non-conception-related activities holds notable advantages for the wellbeing of individuals facing infertility, especially when these activities align with meaningful life goals and values [23,46,47]. However, it is important that the suggestion to use distraction remains sensitive and does not verge into the realm of invalidation or resemble toxic positivity. For example, statements such as “stop moping and do something productive” is clearly not supportive while “just think happy thoughts and see a funny movie or something” is reminiscent of toxic positivity. Instead, engaging in shared activities that align with a person’s values and life goals can be an effective way to offer support. For example, going for a jog together may be meaningful for someone who values fitness, while volunteering together may resonate with those who value community engagement. Our results underscore that women facing infertility often benefit from non-directive support, such as actively listening and offering empathic validation. Participants emphasized that being able to express their feelings openly without receiving advice was incredibly helpful. This aligns with existing research indicating that women with infertility find it easier to discuss their struggles with someone who has experienced similar challenges [48]. This concept holds true across various chronic conditions, such as cancer and HIV, where receiving support from someone who understands the shared experience has proven beneficial [49–51]. Recent findings even demonstrate the positive impact of social support groups for stroke survivors during the COVID-19 pandemic [52].

Our findings reveal the importance of support from those with lived infertility experience, as the category “shared understanding” highlights. Talking to others who had undergone fertility treatment provided a unique source of empathy and validation, making this support particularly meaningful for many participants. Although not everyone has personal experience with infertility, loved ones can play a supportive role by educating themselves about the emotional challenges associated with infertility. Various documentaries and online blogs offer firsthand accounts of the emotional toll that infertility. Knowledge translation efforts from our team have also attempted to improve public knowledge of infertility [53]. Self-education may help individuals avoid unhelpful responses, such as toxic positivity, unsolicited advice, or comments that minimize the distress of infertility—common pitfalls that emerged in the overall category of “unhelpful comments”.

Support for infertility lies in simple, non-intrusive forms of engagement, such as listening without offering solutions or validating the person’s pain. These insights not only highlight the types of support that were most meaningful during treatment suspensions but also emphasize the value of tailoring support to each individual’s unique needs and perspectives.

### Limitations

The current study was conducted amid the COVID-19 pandemic, which may have led women with infertility to be more prone to viewing comments received by others as unhelpful. It is therefore possible that some of the results may not fully generalize to more typical circumstances. Other limitations of the current study include the brief responses provided by participants regarding the comments received by others. A semi-structured interview format, as opposed to a written survey, may have generated additional information regarding the characteristics that differentiate helpful from unhelpful comments. Given that the study included only individuals with sufficient financial means to pursue fertility treatments, a high proportion of our sample was White, educated, and of an elevated socioeconomic status, consistent with other studies of fertility clinic patients [12,54,55]. Had the current study included a more diverse sample, we might have observed additional categories or perhaps differential endorsement of the identified categories. In terms of data analysis, our focus on manifest content analysis—capturing explicit, surface-level content—means that we may have overlooked latent or implicit themes that could provide further insights into participants’ underlying emotions and unspoken experiences. We also chose to report the frequency of content endorsement to highlight commonly shared perspectives. While useful, reporting frequency may inadvertently suggest that more frequently endorsed categories are inherently more significant or meaningful, which could lead to potential misinterpretation This may have also caused subtle but important differences in perspectives to be overlooked.

## Conclusion

In summary, our results suggest that women with infertility find it more helpful when loved ones provide support through active listening, sharing reasons for hope while validating their negative feelings, abstaining from advice-giving, and offering to engage in meaningful, distracting activities together. Loved ones might also consider increasing their ability to empathize by reading or watching first-hand accounts of the emotional toll that infertility can take. While these categories were identified specifically in relation to the COVID-19 pandemic, they are likely largely applicable to non-pandemic times as well. The findings of the current study could serve as a foundation for future knowledge translation campaigns, aimed at raising awareness about the impact of words and actions on women with infertility and emphasizing the crucial role of friends, family, and healthcare professionals in providing helpful support.

## Supporting information

S1 Supplementary File This file contains the steps of providing care for someone experiencing infertility informed by the current study to guide future implementation. (DOCX)

S1 Checklist Strobe checklist. (DOCX)
