# Supplementary material for: What women want to hear: the helpful and unhelpful comments reported by women struggling with infertility amidst the COVID-19 pandemic
Source: PLoS One. 2025 Feb 25;20(2):e0318921. doi: 10.1371/journal.pone.0318921 (PMC11856580; doi:10.1371/journal.pone.0318921)
Supplement: S1 Supplementary File — (DOCX) [file pone.0318921.s001.docx]

S1 Supplementary File

Steps of providing care for individuals with infertility:

1. **Educate Yourself:**
   1. Familiarize yourself with the diverse experiences of infertility through blogs, videos, or documentaries.
   2. Example resources:
      1. Resources & Video: <https://www.letstalkinfertility.org>
      2. Blog: <https://ourmisconception.com>
      3. Documentary: <https://www.youtube.com/watch?v=Wszhtaslvy4>
2. **Caution in Giving Advice:**
   1. Understanding the extensive efforts and research undertaken by individuals with infertility
   2. Evaluate the accuracy of the advice and consider the significance of personal experiences compared to the extensive knowledge of the individual’s medical care team.
3. **Avoid Invalidation:**
   1. Be mindful not to undermine or invalidate the experiences of those facing infertility, recognizing the distress experiences is comparable to other chronic health conditions like cancer.
4. **Respect Personal Space:**
   1. Acknowledge that individuals may need space before they are ready to accept support.
5. **Supporting Techniques**
   1. Consider these reported helpful techniques:
      1. **Listening:**
         1. Provide an empathetic space for those to experience frustrations with tier experience without interruptions or advice.
         2. Utilize active listening skills, including turning off distractions, offering undivided attention, and asking unintrusive follow-up questions.
      2. **Fostering Hope:**
         1. This type of support involves instilling hope in the individual that their difficulty with infertility or the cancellation of treatments are only temporary. This may also involve discussions around ways to find joy in life outside of parenthood. It is important to note that this type of comment is not about instilling false hope or being disingenuous about the likelihood of treatment success.
      3. **Encourage Distraction:**
         1. Suggest engaging in activities outside of attempts to conceive, promoting new experiences to divert attention from fertility challenges.
         2. It can be helpful to encourage activities that may be in line with a person’s values, so if they value personal fitness but have been neglecting this area it may be helpful to suggest going for a walk together.
      4. **Validation:**
         1. Acknowledge the difficulty of their experience without minimizing their feelings.
      5. **Offer of Tangible Support:**
         1. Some appreciate tangible support, such as cooking a meal after a tough week, bringing a treat, financial assistance for fertility treatments.
6. **Adapt Support:**
   1. Continuously assess if your support aligns with the individual’s needs.
   2. Tailor your approach, considering factors like spirituality and culture based on the individual’s needs and beliefs.
7. **Combination of Techniques:**
   1. Recognize that a combination of these techniques may be the most effective support.
   2. It is important to note that active listening is highlighted as one of the most beneficial approaches, so if an individual is unsure of what to say listening may be an important tool.
8. **Avoid unhelpful support styles:**
   1. Toxic Positivity:
      1. This is the tendency to insist that individuals with infertility maintain a positive attitude and avoid all negative emotions.
      2. This can also be related to providing unrealistic outcomes “just relax and it will happen”.
   2. Giving Advice:
      1. Many find receiving advice unhelpful as this advice is frequently devoid of evidence and can be frustrating when individuals spend a great deal of time researching and consulting with their care team (e.g., “I knew someone who X and they got pregnant”).
   3. Invalidation:
      1. This often comes from people lacking an understanding of the experience of infertility and may happen without malice intent (e.g., “it could be worse you could have cancer”).
   4. Intruding:
      1. Be cautious not to ask questions that could be construed as intruding on the individual with infertility (e.g., “when are you going to have a baby”).
9. **Always listen to the feedback of the individual with infertility and adjust.**
