# Supplementary material for: What women want to hear: the helpful and unhelpful comments reported by women struggling with infertility amidst the COVID-19 pandemic
Source: PLoS One. 2025 Feb 25;20(2):e0318921. doi: 10.1371/journal.pone.0318921 (PMC11856580; doi:10.1371/journal.pone.0318921)
Supplement: S1 Checklist — (DOCX) [file pone.0318921.s002.docx]

STROBE Statement—checklist of items that should be included in reports of observational studies

|  | Item No. | Recommendation | Page  No. | Relevant text from manuscript |
| --- | --- | --- | --- | --- |
| **Title and abstract** | 1 | (*a*) Indicate the study’s design with a commonly used term in the title or the abstract | 2 | “…recruited via social media to complete an online survey, which included two open-ended questions” |
|  |  | (*b*) Provide in the abstract an informative and balanced summary of what was done and what was found | 2 | “Eighty women from Canada and the United States (ages 20–45 years) whose fertility treatments had been cancelled due to the COVID-19 pandemic were recruited via social media to complete an online survey, which included two open-ended questions about the most helpful and unhelpful social interactions they had had about their infertility. Two independent researchers conducted content analysis to identify categories of helpful and unhelpful social interactions. The percentage of women endorsing each category was calculated.”  “The following five categories were identified and endorsed by the following percentages of women: 1) Listening (35%), 2) Fostering hope (35%), 3) Talking to individuals with lived experience (13%), 4) Distraction (8%), 5) Validating emotions (6%), and 6) Tangible support (3%). Responses about unsupportive interactions fell into four categories: 1) Toxic positivity (41%), 2) Advice-giving (28%), 3) Invalidation (24%), and 4) Intruding (7%).” |
| Introduction | | | |  |
| Background/rationale | 2 | Explain the scientific background and rationale for the investigation being reported | 3-4 | “In our study on the psychological toll of fertility treatment suspensions, perceived social support was found to be positively associated with mental health amidst treatment suspensions (23). However, that report did not examine the types of comments received by loved ones related to treatment suspensions, though this information was collected in that survey. Specifically, participants were asked to report the helpful comments they had received by loved ones amidst COVID-related treatment suspensions, as well as those experienced as unhelpful.” |
| Objectives | 3 | State specific objectives, including any prespecified hypotheses | 4 | “Specifically, participants were asked to report the helpful comments they had received by loved ones amidst COVID-related treatment suspensions, as well as those experienced as unhelpful. The purpose of the current report is to report on these additional data.” |
| Methods | | | |  |
| Study design | 4 | Present key elements of study design early in the paper | 5-6 | “Participants were asked to complete a survey reporting on coping strategies and the perceived impact of treatment suspensions on their psychosocial wellbeing, the results of which have been previously published (23). The main content of interest for the current report involved participants’ answers to two open-ended questions.” |
| Setting | 5 | Describe the setting, locations, and relevant dates, including periods of recruitment, exposure, follow-up, and data collection | 5-6 | “Women from across Canada and the United States were recruited to participate in the current online study via advertising on Facebook between April 26th and June 13th, 2020, as previously described (23).” |
| Participants | 6 | (*a*) *Cohort study*—Give the eligibility criteria, and the sources and methods of selection of participants. Describe methods of follow-up  *Case-control study*—Give the eligibility criteria, and the sources and methods of case ascertainment and control selection. Give the rationale for the choice of cases and controls  *Cross-sectional study*—Give the eligibility criteria, and the sources and methods of selection of participants | 5 | To qualify, women had to live in Canada or the United States and report having had their fertility treatments suspended due to the COVID-19 pandemic and that their treatments had not yet resumed. The study ad instructed prospective participants to message the research team if they were interested. A member of the team then verified their eligibility to participate.”  “A purposive sampling method was used to reach a large and diverse sample of individuals whose fertility treatments had been cancelled in either Canada or the United States.” |
|  |  | (*b*) *Cohort study*—For matched studies, give matching criteria and number of exposed and unexposed  *Case-control study*—For matched studies, give matching criteria and the number of controls per case | NA |  |
| Variables | 7 | Clearly define all outcomes, exposures, predictors, potential confounders, and effect modifiers. Give diagnostic criteria, if applicable | 6 | “The main content of interest for the current report involved participants’ answers to two open-ended questions: 1) “What were some of the most helpful things people said to you to help you cope with your fertility struggles?” and 2) “What were some of the most unhelpful things people said to you to help you cope with your fertility struggles?”.” |
| Data sources/ measurement | 8* | For each variable of interest, give sources of data and details of methods of assessment (measurement). Describe comparability of assessment methods if there is more than one group | 6 | “Data from the open-ended questions was exported into Excel for data extraction. Content analysis was conducted independently by the first and second authors” |
| Bias | 9 | Describe any efforts to address potential sources of bias | 6 | “The third author helped to resolve disagreements. The interrater reliability of the coding was 93% for the helpful comments and 95% for the unhelpful comments, resulting in an overall interrater reliability of 94%.” |
| Study size | 10 | Explain how the study size was arrived at | 5 | “The target sample size of 100 was chosen to achieve adequate statistical power to detect significant relationships between variables assessed via the quantitative portion of the study (23).” |

Continued on next page

| Quantitative variables | 11 | Explain how quantitative variables were handled in the analyses. If applicable, describe which groupings were chosen and why | 11 | “Chi-squared tests of independence were used to explore the influence of characteristics on participants' endorsement of the identified themes.” |
| --- | --- | --- | --- | --- |
| Statistical methods | 12 | (*a*) Describe all statistical methods, including those used to control for confounding | 6 | “The relationship between participant characteristics and propensity to endorse the identified themes for helpful and unhelpful comments was examined using a Chi-Square test of independence in SPSS (v.27).” |
|  |  | (*b*) Describe any methods used to examine subgroups and interactions |  |  |
|  |  | (*c*) Explain how missing data were addressed | 6 | “Of these 80 participants, six answered only one of the open-ended questions; the answer that they did provide was included in the coding.” |
|  |  | (*d*) *Cohort study*—If applicable, explain how loss to follow-up was addressed  *Case-control study*—If applicable, explain how matching of cases and controls was addressed  *Cross-sectional study*—If applicable, describe analytical methods taking account of sampling strategy | NA |  |
|  |  | (*e*) Describe any sensitivity analyses | NA |  |
| Results | | | | |
| Participants | 13* | (a) Report numbers of individuals at each stage of study—eg numbers potentially eligible, examined for eligibility, confirmed eligible, included in the study, completing follow-up, and analysed | 6 | “Though 92 participants completed the survey, only 80 participants responded to one or both of the open-ended questions on helpful and unhelpful interactions. Of these 80 participants, six answered only one of the open-ended questions; the answer that they did provide was included in the coding.” |
|  |  | (b) Give reasons for non-participation at each stage | NA |  |
|  |  | (c) Consider use of a flow diagram | NA |  |
| Descriptive data | 14* | (a) Give characteristics of study participants (eg demographic, clinical, social) and information on exposures and potential confounders | 7 | “Of participants included in the qualitative analysis, ages ranged from 20 to 45 years, with a mean of M(SD) = 34.2 (4.8). Time spent trying to conceive ranged from 5 to 180 months, with a mean of M(SD) = 37.6 (33.7). In terms of the types of treatments that were canceled, 70% had had an IVF cycle cancelled, and 30% were in the midst of IUI. Seventy-four percent of participants were White, 3% Black, 8% Latina, 13% Asian, and 2% other. Most women were experiencing primary infertility, with 40% of participants having one biological child. Ninety-five percent of participants were married or in a common-law relationship. Fifty-three percent of participants were from Canada, and 47% were from the United States. In terms of the highest level of education achieved, 10% had a high school diploma, 18% had completed some university, 44% held a bachelor’s degree, 21% held a master’s degree, and 8% held a doctorate. Concerning income, 60% of the sample made $90 000 a year or greater.” |
|  |  | (b) Indicate number of participants with missing data for each variable of interest | 9-10 | “n=77” |
|  |  | (c) *Cohort study*—Summarise follow-up time (eg, average and total amount) | NA |  |
| Outcome data | 15* | *Cohort study*—Report numbers of outcome events or summary measures over time | NA |  |
|  |  | *Case-control study—*Report numbers in each exposure category, or summary measures of exposure | NA |  |
|  |  | *Cross-sectional study—*Report numbers of outcome events or summary measures | NA |  |
| Main results | 16 | (*a*) Give unadjusted estimates and, if applicable, confounder-adjusted estimates and their precision (eg, 95% confidence interval). Make clear which confounders were adjusted for and why they were included | NA |  |
|  |  | (*b*) Report category boundaries when continuous variables were categorized | NA |  |
|  |  | (*c*) If relevant, consider translating estimates of relative risk into absolute risk for a meaningful time period | NA |  |

Continued on next page

| Other analyses | 17 | Report other analyses done—eg analyses of subgroups and interactions, and sensitivity analyses | NA |  |
| --- | --- | --- | --- | --- |
| Discussion | | | | |
| Key results | 18 | Summarise key results with reference to study objectives | 13 | “The results of the current study may help serve this need by clearly identifying the types of comments that women with infertility find helpful versus unhelpful in the face of an incredibly challenging situation – that of fertility treatment suspensions related to the COVID-19 pandemic. At the same time, though, results highlight how easy it might be for a well-intentioned loved one to inadvertently say something unhelpful; after all, for the most part, quotes exemplifying unhelpful comments seemed to be well-intentioned.” |
| Limitations | 19 | Discuss limitations of the study, taking into account sources of potential bias or imprecision. Discuss both direction and magnitude of any potential bias | 18-19 | “limitations of the current study include the brief responses provided by participants regarding the comments received by others. A semi-structured interview format, as opposed to a written survey, may have generated additional information regarding the characteristics that differentiate helpful from unhelpful comments.” |
| Interpretation | 20 | Give a cautious overall interpretation of results considering objectives, limitations, multiplicity of analyses, results from similar studies, and other relevant evidence | 13-18 | “In summary, individuals can provide support to their loved ones with infertility through active listening, sharing reasons for hope while validating their negative feelings, abstaining from advice-giving, and offering to engage in meaningful, distracting activities together. Loved ones might also consider increasing their ability to empathize by reading or watching first-hand accounts of the emotional toll that infertility can take. While these themes were identified specifically in relation to the COVID-19 pandemic, they are likely largely applicable to non-pandemic times as well.” |
| Generalisability | 21 | Discuss the generalisability (external validity) of the study results | 17-18 | “It is therefore possible that the prevalence of unhelpful comments received by those experiencing infertility may be higher in the context of the pandemic. Similarly, it is possible that women with infertility, in light of the enormous emotional toll that treatment suspensions has had, are more prone to viewing comments received by others as unhelpful. The results may therefore not fully generalize to more typical circumstances.” |
| Other information | |  | | |
| Funding | 22 | Give the source of funding and the role of the funders for the present study and, if applicable, for the original study on which the present article is based | 1 | “This research was supported by the Saskatchewan Health Research Foundation grant 4577. Dr. Gordon is also supported by a Tier II Canadian Institutes of Health (CIHR) Canada Research Chair and Ashley Balsom is supported by a Social Sciences and Humanities Research Council Doctoral Fellowship.” |

*Give information separately for cases and controls in case-control studies and, if applicable, for exposed and unexposed groups in cohort and cross-sectional studies.

**Note:** An Explanation and Elaboration article discusses each checklist item and gives methodological background and published examples of transparent reporting. The STROBE checklist is best used in conjunction with this article (freely available on the Web sites of PLoS Medicine at http://www.plosmedicine.org/, Annals of Internal Medicine at http://www.annals.org/, and Epidemiology at http://www.epidem.com/). Information on the STROBE Initiative is available at www.strobe-statement.org.
